# Navigating renal safety in the biologic treatment of psoriasis: from immunologic mechanisms to clinical practice

**DOI:** 10.3389/fimmu.2026.1787836

**Published:** 2026-05-01

**Authors:** Zijie Tang, Jintong Wu, Meihan Dong, Chengxin Li, Rui Wang

**Affiliations:** 1 Medical School of Chinese People’s Liberation Army (PLA), Beijing, China; 2 Department of Dermatology, the First Medical Center of Chinese PLA General Hospital, Beijing, China; 3 Department of Dermatology, Central Medical Branch of Chinese PLA General Hospital, Beijing, China; 4 State Key Laboratory of Kidney Diseases, the First Medical Center of Chinese PLA General Hospital, Beijing, China

**Keywords:** biologics, chronic kidney disease, IL-17 inhibitors, psoriasis, renal function, TNF-α

## Abstract

Psoriasis is a common chronic inflammatory skin disorder frequently accompanied by multisystem involvement, with increasing concern regarding its association with renal impairment. The advent and widespread use of biologic agents in the treatment of moderate to severe psoriasis have transformed therapeutic strategies, yet their effects on renal function and the underlying mechanisms remain a critical area of investigation. This review synthesizes current clinical and experimental evidence on the renal impact of biologics used in psoriasis management, focusing on the safety profiles of different biologic classes and their roles in modulating kidney-related complications. We explore how immune regulation, inflammation suppression, and cell death pathways contribute to both renal protection and injury, highlighting the influence of tumor necrosis factor (TNF)-α inhibitors, interleukin (IL) -17 and IL-12/23 inhibitors. By integrating recent clinical studies and basic research findings, this article aims to provide clinicians with a theoretical basis for the rational selection of biologic therapies in psoriasis patients with renal concerns and to stimulate further research into optimizing treatment strategies that safeguard kidney health.

## 1 Introduction

Psoriasis is a chronic inflammatory skin disorder increasingly recognized as a systemic disease with multi-organ involvement, including metabolic syndrome, cardiovascular disease, hypertension, diabetes mellitus, obesity, and chronic kidney disease (CKD) (1). The concept of “psoriatic nephropathy” was first introduced in 2005, highlighting a potential pathological link between psoriasis and renal impairment (2). This association has been substantiated by epidemiological and clinical studies revealing an elevated incidence of CKD and progression to end-stage renal disease (ESRD) in patients with psoriasis, especially those with moderate-to-severe disease phenotypes. The pathophysiology underlying renal involvement in psoriasis is complex, involving systemic inflammation, immune dysregulation, and metabolic derangements that collectively contribute to renal endothelial dysfunction and tissue injury (1, 2).

The advent of biologic therapies targeting key inflammatory cytokines and pathways has revolutionized the management of moderate-to-severe psoriasis. Biologics such as TNF-α inhibitors, IL-17 inhibitors, and IL-12/23 inhibitors have demonstrated significant clinical efficacy by selectively modulating pathogenic immune cascades responsible for psoriatic inflammation (1). These agents have not only improved skin and joint symptoms but also hold promise in mitigating systemic inflammation that may underlie psoriatic comorbidities. However, despite their targeted mechanisms, the impact of biologic therapies on renal function remains incompletely understood. Despite their efficacy, biologic agents raise three categories of concern regarding renal safety: (i) potential direct nephrotoxicity, (ii) altered immune surveillance predisposing to infections that may secondarily affect the kidney, and (iii) an uncertain risk-benefit balance in patients with pre-existing renal impairment or at risk for CKD progression (2, 3). These concerns are compounded by limited longitudinal data on renal outcomes in biologic-treated psoriasis patients.

Recent clinical observations and retrospective analyses have begun to delineate the relationship between biologic therapy and renal function in psoriasis. Preliminary clinical comparisons suggest that biologic therapy is not associated with a higher CKD incidence than conventional systemic therapy such as methotrexate (3). Moreover, case reports have documented successful use of biologics such as adalimumab and secukinumab in psoriatic patients with concomitant ESRD or IgA nephropathy (IgAN), indicating potential safety and efficacy in this vulnerable population (2, 4). Nevertheless, these findings underscore the necessity for vigilant monitoring and multidisciplinary management to balance therapeutic benefits against possible nephrological risks.

Understanding the biological mechanisms linking skin inflammation to kidney pathology is essential. Chronic systemic inflammation driven by cytokines such as TNF-α, IL-17, and IL-36 perpetuates cutaneous lesions while contributing to renal endothelial injury, fibrosis, and functional decline (2, 5). Beyond classical cytokine pathways, emerging molecular mechanisms including ferroptosis of keratinocytes as initiators of systemic inflammatory circuits, which may extend to renal tissues (6). Additionally, post-translational modifications such as RNA methylation and protein glycosylation have been identified as regulators of inflammatory and fibrotic processes in kidney diseases, potentially intersecting with psoriatic pathophysiology (7, 8). Understanding these intricate mechanisms is critical for optimizing biologic therapies to not only control skin disease but also preserve or improve renal function.

Given the rising prevalence of psoriasis and its renal complications, understanding how biologics affect kidney health is essential for guiding treatment selection—especially in patients with existing renal comorbidities or high risk of impairment. Furthermore, insights into the molecular interplay between psoriatic inflammation and renal pathology may reveal novel therapeutic targets and biomarkers for early detection and intervention. Hence, a multidisciplinary approach involving dermatologists, nephrologists, and immunologists is essential to tailor biologic treatment strategies that maximize efficacy while minimizing renal adverse effects (1, 2).

In this review, we systematically examine the current evidence regarding the impact of biologics on renal function in patients with psoriasis. We integrate clinical data, including observational studies and case reports, with emerging basic science research to elucidate the underlying biological mechanisms. Our goal is to provide a comprehensive understanding of how biologic therapies influence kidney health in psoriatic patients, thereby informing clinical decision-making and fostering the development of optimized, individualized treatment paradigms that address both cutaneous and systemic disease aspects.

## 2 Clinical correlation between psoriasis and kidney injury

Psoriasis is increasingly recognized as a systemic disease with multi-organ involvement, including the kidneys (9). Epidemiological studies consistently demonstrate that renal lesions represent a notable comorbidity, reflecting the systemic inflammatory burden of psoriasis. While reported incidence varies, a clear trend indicates elevated CKD risk compared to the general population. A cohort study of 219 psoriatic patients found that 17.35% had moderate, 5.02% high, and 3.66% very high CKD risk, with microalbuminuria correlating positively with disease duration, severity, and psoriatic arthritis (10). A hospital-based cross-sectional study identified CKD in 13 of 104 psoriasis patients (12.5%), predominantly those with severe and long-standing disease (11). A meta-analysis encompassing over 700,000 psoriasis patients demonstrated that individuals with psoriasis have a significantly elevated risk of CKD and ESRD compared to non-psoriatic controls, with pooled hazard ratios (HR) of 1.65 for CKD and 1.37 for ESRD (12). Notably, this risk correlates positively with the severity of psoriasis, indicating that patients with more extensive skin involvement are at greater risk for renal impairment (12). This elevated risk stems from persistent systemic inflammation, immune dysregulation, and common comorbidities including hypertension, diabetes, and metabolic syndrome (9, 13). Patients with severe psoriasis or psoriatic arthritis exhibit significantly increased frequency of CKD and related complications, underscoring the importance of renal monitoring (13–15). Moreover, genetic studies using Mendelian randomization have provided evidence supporting a causal relationship between psoriasis and CKD, highlighting that genetically predicted psoriasis increases CKD risk (16).

Importantly, psoriasis patients with CKD often present with comorbidities such as diabetes mellitus and hypertension, which independently contribute to renal impairment and may have additive or synergistic effects on kidney function deterioration. For example, diabetes and hypertension are well-established risk factors for CKD progression and ESRD, and their coexistence with psoriasis exacerbates renal outcomes (11, 17) The presence of these comorbidities necessitates a comprehensive approach to patient management, addressing not only skin disease but also cardiovascular and metabolic health to mitigate renal risk. In summary, robust clinical and genetic evidence supports that psoriasis is an independent risk factor for CKD and ESRD, with severity and duration of skin disease playing critical roles. The coexistence of diabetes, hypertension, and other metabolic comorbidities amplifies the risk and accelerates renal function decline. These findings highlight the need for vigilant renal monitoring and integrated management strategies in patients with psoriasis to prevent progression to advanced kidney disease.

The concept of psoriatic nephropathy has emerged to describe the spectrum of kidney diseases directly or indirectly associated with psoriasis (18). Psoriatic nephropaAthy encompasses a range of renal pathologies, including glomerulonephritis, nephrotic syndrome, and tubulointerstitial nephritis, often mediated by immune complex deposition and chronic inflammation (18). Clinically, patients may present with proteinuria, hematuria, hypertension, and progressive decline in renal function (18). However, the manifestations can be subtle and easily overlooked, necessitating vigilant screening. The pathophysiological mechanisms underlying psoriatic nephropathy may involve complex interactions between systemic inflammatory mediators such as IL-17, IL-23, and TNF-α, which are central to psoriasis pathogenesis and also contribute to renal tissue injury (19). Moreover, genetic predispositions and shared inflammatory pathways with other autoimmune diseases like systemic lupus erythematosus further complicate the clinical picture and may influence the renal outcomes in psoriasis patients (19).

Epidemiological trends show increasing detection rates of renal comorbidities, partly due to enhanced screening and improved recognition of psoriasis as a systemic disease. The advent of biologic therapies targeting inflammatory pathways has raised important questions regarding their impact on renal health, including both potential nephrotoxicity and renal protection. Overall, epidemiological evidence underscores the necessity for integrated management approaches addressing both cutaneous and renal aspects of psoriasis to mitigate complications and improve long-term prognosis.

## 3 Types of biologics for psoriasis and their renal safety

### 3.1 Renal effects of TNF-α inhibitors

#### 3.1.1 Renal safety of TNF-α inhibitors: clinical evidence

TNF-α inhibitors have revolutionized the management of autoimmune diseases such as psoriasis, yet their renal safety profile remains complex. While generally beneficial, case reports have documented uncommon but notable renal adverse events. Development of IgAN has been reported in patients with rheumatoid arthritis or Crohn’s disease during adalimumab therapy, presenting with hematuria, proteinuria, and occasional progression to ESRD (20–22). Drug discontinuation and immunosuppressive therapy often lead to renal recovery, suggesting a paradoxical autoimmune mechanism triggered by TNF-α blockade. Additional pathologies, including diffuse proliferative glomerulonephritis and acute tubulointerstitial nephritis, have also been confirmed by biopsy in adalimumab-treated patients (23). It should be noted that these findings are derived from case reports and small series, which are valuable for signal detection but cannot establish causality or incidence. Larger controlled studies are needed to validate these observations.

Beyond glomerular diseases, tubulointerstitial nephritis (TIN) has also been reported in the context of TNF-α inhibitor therapy, although the etiological attribution remains challenging due to confounding factors such as concomitant medications and underlying inflammatory bowel disease activity (24). In pediatric populations, adalimumab-associated acute kidney injury has been documented, with variable outcomes depending on the timeliness of drug withdrawal and initiation of corticosteroid therapy (25). Importantly, these nephrotoxic events appear rare relative to the widespread use of TNF-α inhibitors, but their recognition is critical for early diagnosis and management.

The safety profile of TNF-α inhibitors in patients with pre-existing renal impairment has also been evaluated. Studies in ankylosing spondylitis patients with CKD demonstrate that anti-TNF therapy, including adalimumab, can be effective and generally safe, with significant improvements in disease activity indices and no substantial deterioration in renal function over follow-up periods (26). Similarly, in solid organ transplant recipients with inflammatory conditions, biologics including adalimumab have been used without a marked increase in serious infections or mortality, although vigilance for infectious and immunological complications remains warranted (27).

#### 3.1.2 Efficacy and safety of adalimumab in psoriasis patients with renal dysfunction

Adalimumab is widely used in moderate to severe psoriasis, with accumulating evidence suggesting its efficacy and safety in patients with renal dysfunction, including those with CKD and ESRD. A case report of a patient with severe plaque psoriasis complicated by ESRD showed that adalimumab treatment led to significant improvement of cutaneous symptoms without exacerbation of renal disease (2). Favorable outcomes have also been reported in pediatric tubulointerstitial nephritis and uveitis (TINU) syndrome, where adalimumab achieved sustained remission (28). Real-world post-marketing surveillance data from patients with hidradenitis suppurativa further support its safety profile (29), and proteomic analyses from clinical trials suggest potential cardiovascular and renal benefits beyond skin control (30).

However, these findings derive primarily from case reports and small series. While valuable for signal detection, such evidence cannot establish definitive conclusions regarding efficacy or safety in broader populations. Rare but severe adverse events have been documented, including antiphospholipid syndrome, diffuse glomerulonephritis, and acute kidney injury, with outcomes ranging from irreversible damage to full recovery upon drug withdrawal and steroid therapy (23, 25). These cases underscore the importance of regular renal function monitoring during adalimumab therapy.

In conclusion, adalimumab appears effective and generally safe for most psoriasis patients with renal impairment. Nevertheless, given the limitations of available evidence, vigilance for rare serious adverse events remains essential. Individualized risk assessment, close monitoring of renal parameters, and prompt management are critical. Larger controlled studies are warranted to confirm these observations and guide clinical practice.

### 3.2 Impact of IL-17 inhibitors on renal function

IL-17 inhibitors, particularly secukinumab, have demonstrated promising clinical outcomes in patients with psoriasis complicated by renal dysfunction, including those with ESRD. A notable case report described a patient with erythrodermic psoriasis undergoing hemodialysis for ESRD who was treated with secukinumab (31). The treatment rapidly alleviated severe skin symptoms and, importantly, was associated with a certain degree of renal function improvement. Over a 7-month follow-up period, the patient maintained clearance of skin lesions without further deterioration of kidney function, an outcome rarely documented in the literature for this patient population (31). This clinical observation suggests that IL-17 blockade may be a favorable therapeutic option for patients with psoriasis and concomitant kidney disease, offering both dermatologic and renal benefits.

Another clinical case involved a patient with systemic sclerosis and ESRD who underwent autologous hematopoietic stem cell transplantation. The patient experienced early skin disease progression post-transplant, which was successfully managed with secukinumab, highlighting the drug’s efficacy even in complex clinical scenarios involving severe renal impairment (32). These clinical observations collectively underscore the potential of IL-17 inhibitors to stabilize or improve renal function in patients with inflammatory conditions complicated by kidney disease, although larger-scale studies are needed to confirm these findings and to define optimal treatment protocols.

### 3.3 IL-12/23 inhibitors: renal safety and therapeutic potential

IL-12/23 inhibitors, targeting the shared p40 subunit of IL-12 and IL-23, have emerged as important biologic agents in the treatment of immune-mediated diseases such as psoriasis, psoriatic arthritis, and certain forms of glomerulonephritis. Their impact on renal function and safety profile in patients with kidney involvement has garnered increasing attention. Clinical data indicate that IL-12/23 inhibitors, such as ustekinumab, exhibit a generally favorable renal safety profile in patients with psoriasis, including those with pre-existing CKD. A retrospective study analyzing the real-world effects of biologics on renal function in psoriatic patients found that treatment with anti-IL-12/23 agents did not significantly worsen estimated glomerular filtration rate (eGFR) or promote progression of CKD stages over a two-year period, with most patients maintaining stable renal function (33).

Moreover, in the context of autoimmune kidney diseases, IL-12/23 inhibition has shown therapeutic promise. For instance, in ANCA-associated glomerulonephritis, a severe form of autoimmune kidney inflammation, immune profiling identified pathogenic T cells producing IL-12 and IL-23 as key drivers of disease activity. Treatment with ustekinumab combined with low-dose immunosuppressants, was well tolerated and led to clinical improvement, including enhanced kidney function and reduced vasculitis activity scores (34). This highlights the potential of IL-12/23 blockade to modulate pathogenic immune responses directly within the kidney microenvironment. Mechanistically, IL-12 and IL-23 are pivotal in promoting Th1 and Th17 immune responses, respectively, which are implicated in renal inflammation and fibrosis. Elevated levels of IL-12 family cytokines, including IL-12p70 and IL-23, have been associated with progression of renal diseases such as autosomal dominant polycystic kidney disease (ADPKD), where increased secretion correlates with declining eGFR and worsening albuminuria (35).

These findings underscore the role of IL-12/23 pathways in kidney disease pathogenesis and provide a rationale for targeted intervention. Additionally, preclinical studies have demonstrated that IL-12 and IL-23 subunits are upregulated in immune cells responding to kidney injury and infection, contributing to inflammatory cascades (36). The modulation of these cytokines may therefore attenuate renal inflammation and subsequent damage. Importantly, safety considerations for IL-12/23 inhibitors include monitoring for infections and malignancies, as highlighted in regulatory warnings; however, kidney-specific adverse effects appear limited compared to other biologics (37). Taken together, current clinical and translational evidence supports the renal safety of IL-12/23 inhibitors and suggests their therapeutic potential in immune-mediated kidney diseases, warranting further clinical trials to define their role in nephrology.

## 4 Mechanisms of biologic therapy on renal function in psoriasis

The association between psoriasis and CKD is mediated through systemic inflammation-driven pathogenic mechanisms. Understanding these mechanisms is essential for comprehending how biologic therapies may confer renal protection.

### 4.1 Pathogenic mechanisms linking psoriasis to kidney injury

Psoriasis is a chronic immune-mediated inflammatory disease characterized by dysregulated cytokine networks and immune cell activation, contributing to remote organ damage including the kidneys (9). Accumulating evidence suggests a potential link between psoriasis and IgAN, indicating shared immunopathological mechanisms (38).

The immune-mediated inflammatory response is central to kidney injury in psoriasis. Immune dysregulation triggers a cascade of events leading to renal tissue damage. Infiltration of T lymphocytes, macrophages, and dendritic cells into the renal parenchyma initiates and sustains local inflammation. These cells release proinflammatory cytokines and chemokines that promote endothelial dysfunction, mesangial proliferation, and tubular injury, ultimately impairing renal function. Among these mediators, IL-17 and TNF-α are pivotal. IL-17, primarily produced by Th17 cells, enhances neutrophil recruitment and activation, upregulates other inflammatory cytokines, and disrupts the pro-/anti-inflammatory balance in the kidney microenvironment (39). Besides, TNF-α exacerbates renal damage by inducing apoptosis, oxidative stress, and fibrosis through activation of nuclear factor-kappa B (NF-κB) and other downstream pathways (40). The interplay between IL-17 and TNF-α creates a vicious cycle of sustained inflammation and tissue injury. Furthermore, transcription factors such as FOXM1 have been implicated in modulating these immune responses. FOXM1 regulates genes involved in cell proliferation, apoptosis, and inflammation, and its dysregulation may contribute to the progression of kidney injury in psoriasis by influencing inflammatory signaling pathways including NF-κB (41).

Shared immune-related gene signatures and increased T-cell infiltration in both psoriatic and CKD tissues suggest that immune dysregulation and chronic systemic inflammation link psoriasis to kidney disease (42). Animal models further demonstrate that granulocyte colony-stimulating factor (G-CSF) mediates renal neutrophil accumulation and damage in psoriasis, offering mechanistic insight (43).

In addition to direct inflammatory effects, chronic inflammation affects kidney function through systemic mechanisms. Chronic low-grade inflammation in CKD patients is associated with increased cardiovascular risk, which complicates renal disease management (44). Cardiovascular disease can lead to renal hypoperfusion and ischemia, creating a feedback loop that exacerbates renal dysfunction. Additionally, systemic inflammation can activate the renin-angiotensin-aldosterone system (RAAS), contributing to hypertension and fluid retention, thereby further impairing renal function (45).

### 4.2 Renoprotective mechanisms of biologic therapy

Biologic agents used in psoriasis treatment may confer renal protection through suppression of inflammatory cascades that underlie both cutaneous and systemic disease.

#### 4.2.1 Anti-inflammatory effects

Mechanistically, TNF-α inhibition may modulate renal inflammation and fibrosis pathways, as suggested by animal studies where TNF-α blockade infliximab attenuated renal injury induced by combined insults such as periodontitis and high phosphate intake (46).

The beneficial effects of IL-17 inhibitors on renal function are likely mediated through their capacity to suppress IL-17-driven inflammatory pathways that contribute to kidney injury. Experimental studies have demonstrated that, in ischemia-reperfusion injury models, increased IL-17 expression correlates with enhanced phosphorylation of NF-κB pathway components, leading to amplified inflammatory responses and tubular epithelial cell apoptosis. Deficiency of aldehyde dehydrogenase 2 exacerbates this process by promoting IκBα/NF-κB p65 phosphorylation and increasing IL-17C expression, thereby aggravating renal injury (47).

The role of IL-17 in kidney inflammation is further supported by studies showing that modulation of Th17/Treg balance, which regulates IL-17 production, significantly impacts renal inflammation and injury outcomes. For example, miR-126 was found to reduce Th17 differentiation and IL-17 levels, leading to decreased inflammatory infiltration and improved kidney histopathology in septic rats (48). The IL-17 pathway’s involvement in renal inflammation is also evident in cholestasis-induced kidney injury, where NF-κB-mediated inflammatory responses, including IL-17 upregulation, contribute to tissue fibrosis and functional impairment; inhibition of NF-κB signaling ameliorated these effects (49). By targeting IL-17, secukinumab and other IL-17 inhibitors likely reduce recruitment and activation of inflammatory cells, decrease pro-inflammatory cytokine production, and attenuate downstream signaling pathways such as NF-κB and MAPK within the kidney. This immunomodulatory effect can stabilize or improve renal function by limiting ongoing inflammation, preventing fibrosis, and promoting tissue repair. Moreover, IL-17 blockade may also influence macrophage polarization and other immune cell functions, further contributing to the suppression of renal inflammation (50).

IL-23 inhibitors suppress Th17 differentiation, thereby reducing downstream IL-17 production. The JAK-STAT pathway represents an additional therapeutic target, as its inhibition may decrease galactose-deficient IgA1(Gd-IgA1) production in IgAN (51). Inhibition of NF-κB, MAPKs, and STAT3 pathways reduces pro-inflammatory gene expression and fibrogenesis (52, 53). By targeting these upstream inflammatory mediators, biologic agents may prevent or attenuate renal inflammation and fibrosis.

#### 4.2.2 Cell death pathways in kidney injury

Pyroptosis, a form of proinflammatory programmed cell death characterized by cell swelling, membrane rupture, and release of inflammatory cytokines, has emerged as a critical mechanism in the pathogenesis of various kidney injuries. Recent studies have highlighted the pivotal role of pyroptosis in acute kidney injury (AKI), CKD, diabetic nephropathy (DN), and other renal disorders. Unlike apoptosis or necrosis, pyroptosis is mediated primarily by the cleavage of gasdermin proteins (notably gasdermin D and E) following inflammasome activation and caspase cleavage, leading to pore formation in the plasma membrane and subsequent inflammatory cell death (54, 55). In AKI, tubular epithelial cells undergo pyroptosis triggered by inflammasome activation, particularly the NLRP3 inflammasome, which results in the release of proinflammatory cytokines such as IL-1β and IL-18, exacerbating renal inflammation and injury (56, 57). The involvement of pyroptosis has also been demonstrated in calcium oxalate crystal-induced kidney injury, where gasdermin D-mediated pyroptosis contributes to tubular damage and stone formation, highlighting the interplay between cell death and renal pathology (58, 59). Furthermore, the role of pyroptosis extends to drug-induced nephrotoxicity, where pyroptotic pathways mediate tubular cell death and inflammation, limiting the clinical use of someagents (60, 61). The crosstalk between pyroptosis and other regulated cell death forms, including ferroptosis and necroptosis, further complicates the pathophysiological landscape but offers multiple therapeutic targets (62). In the context of inflammatory kidney diseases, pyroptosis contributes to podocyte injury in DN and membranous nephropathy, linking inflammasome activation and mitochondrial dysfunction to glomerular pathology (63, 64).

Biologic therapies targeting key inflammatory cytokines and signaling pathways in psoriasis may exert renoprotective effects by modulating pyroptosis and associated molecular mechanisms. Biologics such as TNF-α inhibitors, IL-17 and IL-23 antagonists, widely used in psoriasis treatment, reduce systemic inflammation and downregulate inflammasome activation, which is central to pyroptosis (55, 56). By attenuating upstream signals that trigger NLRP3 inflammasome assembly and caspase activation, biologics can potentially inhibit gasdermin-mediated pyroptotic cell death in renal tubular cells, thereby mitigating kidney injury. Experimental studies have demonstrated that pharmacological agents targeting inflammasome components or caspases reduce pyroptosis and improve renal outcomes in AKI and CKD models (55, 65). Additionally, compounds with antioxidative properties, such as quercetin and carnosol, have been shown to inhibit pyroptosis by reducing oxidative stress and inflammasome activation, suggesting that biologics with anti-inflammatory and antioxidative effects might similarly modulate these pathways (66, 67). Mechanistically, biologics may interfere with signaling cascades such as the DPP4-JNK pathway, PI3K/AKT/NRF2 axis, and TSPO-mediated macrophage pyroptosis, which have been implicated in renal pyroptosis regulation (68–70).

**Figure 1:**
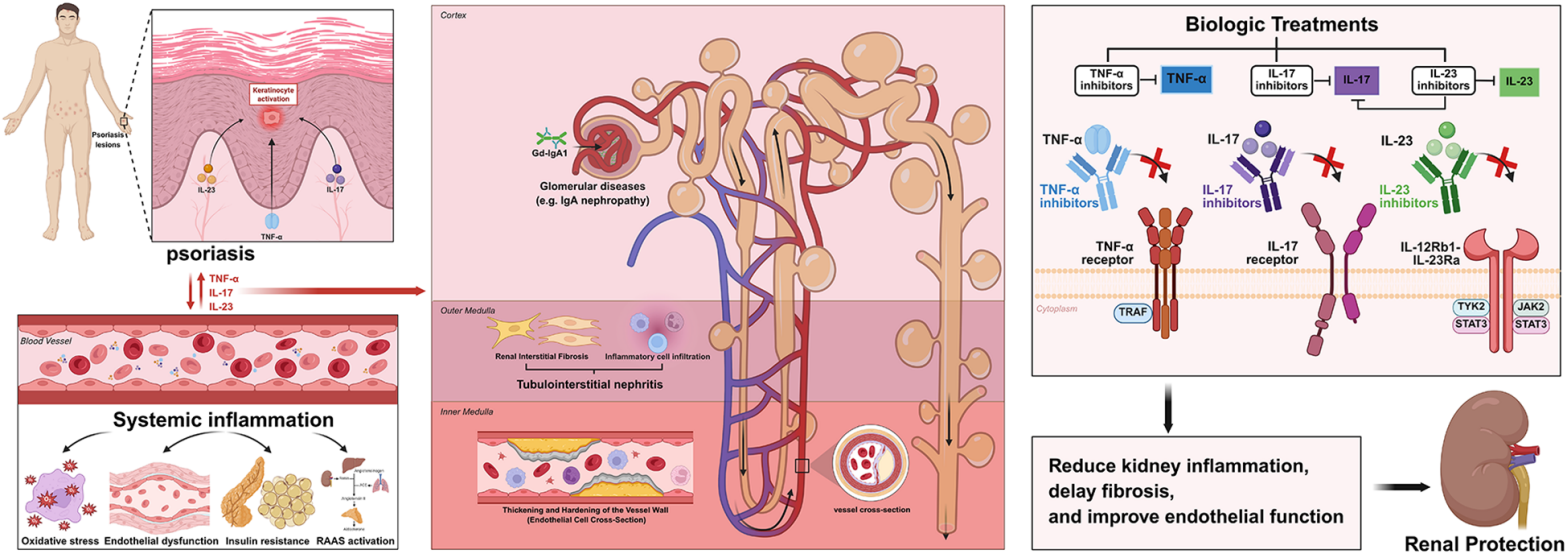
Immune crosstalk and mechanisms between psoriasis and CKD, in which systemic inflammation plays a key role, then disrupted by various biologics. This figure illustrates the pathophysiology of inflammatory cytokines derived from skin and blood on different anatomical parts of the kidney, as well as the actions of biologics on renal protection.

**Figure 2:**
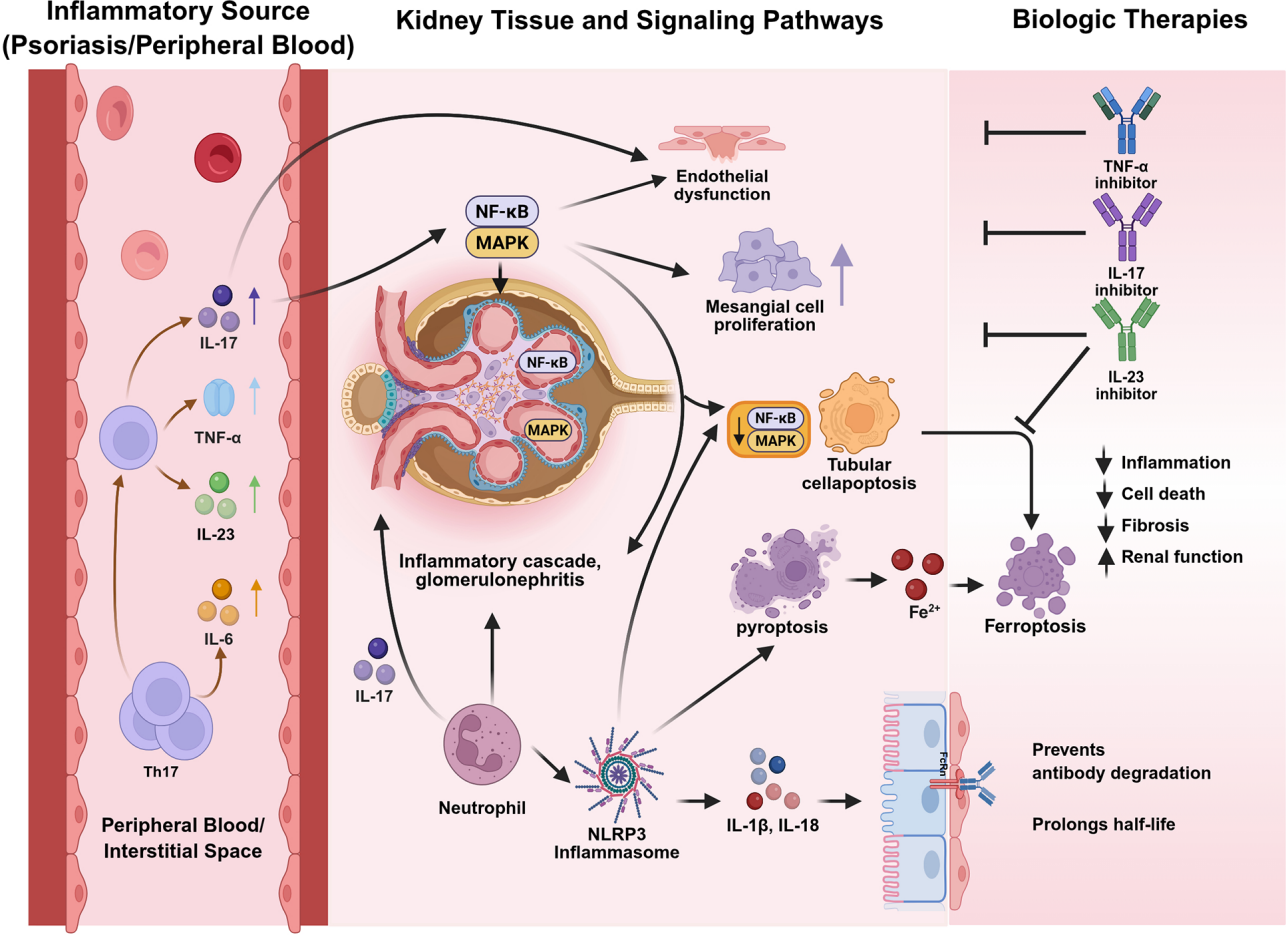
Integrated mechanisms of biologic-induced renal protection in psoriasis. Biologics exert renal protective effects via three interconnected pathways: (1) suppression of NF-κB/MAPK-driven inflammation; (2) inhibition of NLRP3/caspase-1/gasdermin-mediated pyroptosis; and (3) expression of the neonatal Fc receptor (FcRn) in endothelial cells binds to the Fc portion of IgG. FcRn modulates renal drug clearance and exposure.

In the myocardial remodeling model, IL-23p19 knockout can attenuate macrophage ferroptosis and improve cardiac function (71). This suggests that the IL-23 pathway itself may promote ferroptosis. IL-23 is a key cytokine for Th17 cell differentiation, and inhibiting IL-23 theoretically reduces the Th17/IL-17-driven inflammatory cascade. Thus, ferroptosis can amplify the inflammatory response, while IL-23 inhibitors may indirectly alleviate ferroptosis-related tissue damage while reducing inflammation. Although direct clinical evidence linking biologics used in psoriasis to modulation of renal pyroptosis is limited, the convergence of inflammatory pathways suggests that biologics may confer renal protection by inhibiting pyroptosis and its downstream inflammatory sequelae (**Figures 1**, **2**).

### 4.3 Potential impact of FcRn receptor and biologics pharmacokinetics on the kidney

The neonatal Fc receptor (FcRn) plays a pivotal role in the pharmacokinetics of Fc-containing biologics, influencing their distribution and clearance in various tissues, including the kidney. FcRn is responsible for protecting IgG antibodies and Fc-fusion proteins from lysosomal degradation by binding them at acidic pH within endosomes and recycling them back to the cell surface, thereby prolonging their half-life in circulation. This receptor-mediated salvage pathway is critical for maintaining systemic levels of therapeutic antibodies used in psoriasis treatment, such as biologics targeting immune pathways. Quantitative proteomic analysis has shown that FcRn expression varies significantly among human tissues, with the highest abundance of the FcRn p51 subunit observed in the liver, followed by the kidney, intestine, and skin (72). The β2-microglobulin (B2M) subunit, which forms part of the FcRn complex, is generally more abundant than FcRn p51 across tissues, indicating a complex regulation of receptor availability and function. This tissue-specific expression pattern suggests differential capacity for FcRn-mediated recycling and clearance of biologics, influencing their pharmacokinetic profiles and therapeutic efficacy. In the kidney, FcRn expression facilitates the reabsorption and recycling of filtered IgG and Fc-containing biologics, thereby modulating their renal clearance and systemic exposure (72). This mechanism is particularly relevant in the context of biologics used to treat psoriasis, where sustained drug levels are necessary for disease control. Understanding the role of FcRn in biologics disposition is essential for optimizing dosing regimens and minimizing potential nephrotoxicity associated with drug accumulation or altered clearance in renal tissues (72).

Variations in FcRn expression can significantly influence the exposure of the kidney to biologics, with potential implications for both efficacy and toxicity. Studies have demonstrated that FcRn expression is dynamic and can be affected by physiological factors such as age, body mass index (BMI), and disease states (72). For example, FcRn p51 subunit levels are higher in neonatal liver compared to adults, reflecting developmental regulation that may similarly affect renal FcRn expression and function (72). In adult populations, moderate correlations between BMI and FcRn expression in liver and kidney tissues suggest that metabolic status could modulate biologics pharmacokinetics (72). Moreover, pathological conditions such as cancer and psoriasis may alter FcRn abundance in tissues adjacent to disease sites, potentially affecting local drug disposition. In the kidney, changes in FcRn expression could modify the balance between biologic recycling and degradation, influencing drug accumulation within renal cells and interstitial spaces. This altered exposure may impact the kidney’s susceptibility to biologic-induced adverse effects or modify therapeutic outcomes. Physiologically-based pharmacokinetic modeling incorporating FcRn abundance and endogenous IgG levels has successfully predicted biologics disposition in healthy and diseased states, underscoring the receptor’s critical role as a systems parameter. These models reveal that endogenous IgG concentration and tissue FcRn expression are key determinants of biologic exposure, highlighting the importance of considering FcRn variability when evaluating renal pharmacokinetics and safety profiles of biologics used in psoriasis treatment.

## 5 Clinical observation of renal adverse events in psoriasis patients treated with biologics

### 5.1 Renal function stability and studies on the use of biologics

The stability of renal function in patients undergoing biologic therapy, particularly for chronic inflammatory diseases such as psoriasis and ankylosing spondylitis (AS), has been a subject of increasing clinical interest. Large-scale retrospective and prospective studies have provided valuable insights into how different classes of biologics influence kidney function over time. For instance, a retrospective study analyzing data from 601 patients with severe psoriasis treated continuously with biologics for at least two years found no significant differences in eGFR or progression of CKD stages before and after biologic therapy, regardless of the biologic class used (33). Notably, all patients treated with anti-IL-17 biologics maintained stable CKD stages, suggesting a potentially favorable renal safety profile for this class. Progression of CKD was more closely associated with baseline renal impairment, older age, diabetes, and dyslipidemia, with diabetes identified as an independent risk factor for renal deterioration during biologic treatment. This underscores the importance of patient comorbidities in renal outcomes rather than biologic therapy per se. Complementing these findings, a prospective cohort study involving 211 AS patients treated with anti-TNF agents demonstrated a small but statistically significant decrease in eGFR over time; however, after adjusting for confounders such as disease activity and lifestyle factors, this decline was not clinically relevant, supporting the renal safety of anti-TNF biologics even in patients with pre-existing risk factors for renal decline (73). Moreover, another study focusing on moderate-to-severe chronic plaque psoriasis patients treated with biologics observed a significant reduction in serum creatinine levels after one year of therapy, with improvements in renal function correlating with the degree of psoriasis remission, suggesting that the anti-inflammatory effects of biologics may indirectly benefit renal function by reducing systemic inflammation (74). These clinical data collectively suggest that biologic therapies do not exacerbate renal dysfunction and may, in some cases, contribute to renal function stabilization or improvement by mitigating systemic inflammatory burden. However, the impact of biologics on renal function may vary depending on the specific agent used and patient characteristics, necessitating individualized monitoring. In addition to clinical observations, mechanistic insights from animal models reveal that biological renal support can attenuate AKI by suppressing complement activation, oxidative stress, inflammation, and cell death, while promoting tubular cell proliferation, highlighting potential pathways through which biologics might exert renal protective effects. Furthermore, emerging research emphasizes the role of biological aging and molecular markers such as α-Klotho in renal function decline, suggesting that biologics’ effects on systemic inflammation and aging processes could indirectly influence renal outcomes (75). Taken together, Biologic therapies maintain renal function stability in patients with inflammatory diseases when carefully selected and monitored. Diabetes and baseline renal impairment are key determinants of renal prognosis. These findings support the continued use of biologics without undue concern for renal deterioration, while emphasizing the importance of monitoring renal function and comorbidities.

### 5.2 Incidence and risk factors of kidney-related adverse events

Kidney-related adverse events (AEs), including renal function deterioration and AKI, are critical concerns in the management of psoriasis patients undergoing biologic therapy. The incidence of these renal AEs appears relatively low but clinically significant, necessitating careful monitoring. An observational study involving 6,294 adults with psoriasis or psoriatic arthritis initiating methotrexate (MTX) or biologics found that CKD incidence did not significantly differ between MTX and biologics users, affecting approximately 1.5% of patients over five years, with adjusted HR close to unity (HR = 1.03) indicating similar renal safety profiles between these therapies. Additionally, acute kidney injury and serious infections were rare and showed no meaningful difference between treatment groups, suggesting biologics do not increase AKI risk compared to MTX in routine clinical practice (3). Complementing these findings, a retrospective study of 601 severe psoriasis patients treated continuously with biologics for at least two years demonstrated stable eGFR and CKD staging across anti-TNF, anti-IL-12/23, and anti-IL-17 agents. Only a small subset (2.2%) experienced CKD progression, with diabetes identified as an independent risk factor for renal function deterioration during biologic therapy. Notably, all patients receiving anti-IL-17 biologics maintained stable CKD stages, underscoring a potentially favorable renal safety profile for this class (33).

Age and comorbidities such as diabetes mellitus and pre-existing CKD emerge as important risk factors influencing kidney-related adverse events during biologic treatment. The presence of diabetes significantly increases the risk of renal function decline, consistent with its known role in microvascular damage and CKD progression. Similarly, older age correlates with higher vulnerability to renal impairment, likely due to age-related nephron loss and diminished renal reserve. Another nested case-control study focusing on patients with rheumatic diseases treated with biologic disease-modifying anti-rheumatic drugs (DMARDs) revealed that chronic kidney disease strongly predicts loss of hepatitis B virus surface antibody (anti-HBs), indicating impaired immune status and potential for viral reactivation in this subgroup. This study also highlighted diabetes as a significant risk factor for anti-HBs loss, reinforcing the interplay between metabolic comorbidities and renal impairment in patients receiving biologics (76). The impact of declining kidney function on serious adverse drug reactions (ADRs) further emphasizes the clinical importance of renal status. A prospective CKD cohort study reported that lower eGFR is a major risk factor for serious ADRs, including drug-induced AKI and bleeding events, which frequently necessitate hospitalization and can be life-threatening. Each 1 mL/min/1.73 m^2 decrease in eGFR was associated with a 2.2% increase in AKI risk and an 8% increase in bleeding risk, underscoring the need for vigilant renal function monitoring during biologic therapy, especially in patients with moderate to advanced CKD (77).

Moreover, incidental renal findings such as renal cysts and urolithiasis are more prevalent in psoriasis patients on biologic therapy compared to healthy controls, as demonstrated by computed tomography (CT) imaging studies. Although these findings may not directly translate to acute renal injury, they reflect underlying renal structural alterations or comorbidities that could predispose to renal complications (78). Case reports also provide valuable clinical insights; for instance, a patient with severe psoriasis complicated by IgAN and ESRD on hemodialysis was successfully treated with secukinumab, an IL-17A inhibitor, achieving complete resolution of psoriatic symptoms without renal parameter deterioration over one year (4). This suggests that certain biologics may be safely administered even in advanced kidney disease, although larger studies are needed to establish evidence-based guidelines for biologic selection in this vulnerable population (4).

In summary, kidney-related adverse events during biologic therapy for psoriasis are uncommon but clinically relevant, with incidence rates of CKD and AKI remaining low and comparable to conventional treatments such as MTX. Age, diabetes, and baseline CKD stage are key risk factors for renal function deterioration and adverse outcomes. Careful patient selection, regular renal function monitoring, and multidisciplinary management are essential to mitigate these risks. The current evidence supports the renal safety of biologics, particularly anti-IL-17 agents, but underscores the necessity of individualized risk assessment to optimize therapeutic outcomes while minimizing kidney-related complications.

### 5.3 Application and renal safety of biologics in transplant patients

The use of biologic agents for treating psoriasis and other autoimmune or inflammatory conditions in solid organ transplant (SOT) recipients, including kidney transplant patients, presents unique challenges and considerations due to the underlying immunosuppressive state and risk of complications. Clinical evidence from multiple studies and case series indicates that biologics, particularly anti-TNF agents, anti-integrins, and IL-12/23 inhibitors such as ustekinumab, can be used with relative safety in SOT recipients, including those with kidney transplants, but careful monitoring is essential. For instance, a systematic review encompassing 187 SOT recipients treated with biologics for inflammatory diseases found that anti-TNF agents were the most commonly used biologics and generally did not result in major safety issues, although infections and malignancies were observed in some cases (79). Specifically, kidney transplant recipients on calcineurin inhibitors like cyclosporine or tacrolimus tolerated concomitant biologic therapy well, with only rare reports of malignancy or infection, suggesting a manageable safety profile when biologics are added to standard immunosuppression (80).

However, the immunosuppressed status of transplant recipients necessitates special considerations. The combined immunosuppressive burden from maintenance drugs and biologics increases susceptibility to infections, including opportunistic pathogens, and may elevate malignancy risk. A multicenter retrospective study of rheumatic patients receiving biologic DMARDs post-kidney transplantation reported a high incidence of severe infections, predominantly urinary tract infections and pneumonia, with an infection-related mortality rate of 11% (81). This underscores the importance of individualized risk assessment and vigilant infection surveillance when biologics are prescribed in this population.

Case reports further illustrate the therapeutic potential and safety of biologics in transplant recipients with concurrent autoimmune diseases. For example, tocilizumab, an anti-IL-6 receptor monoclonal antibody, was safely and effectively used in a rheumatoid arthritis patient on maintenance hemodialysis, who was also a kidney transplant candidate, achieving sustained remission without adverse events over four years (82). Similarly, adalimumab successfully treated a kidney transplant recipient with severe psoriasis refractory to conventional therapies, allowing discontinuation of mycophenolate mofetil and demonstrating clinical benefit without reported complications (83).

In the context of transplantation, the pharmacokinetics of biologics may be altered by concomitant therapies. For example, intravenous immunoglobulin administration can accelerate the clearance of monoclonal antibodies such as tesidolumab, potentially reducing their efficacy (84). This interaction highlights the need for careful therapeutic drug monitoring and dose adjustments when biologics are combined with other immunomodulatory agents in transplant recipients.

Moreover, the timing and choice of biologic therapy must consider the transplant type and immunosuppressive regimen. While most data derive from liver and kidney transplant recipients, evidence in heart transplant patients remains limited, necessitating further research (27). The risk-benefit balance of biologic use in transplant recipients is complex, as these agents may help control autoimmune disease activity but increase infection risk and potentially impact graft survival.

To summarize biologic therapies can be cautiously applied in solid organ transplant recipients, including kidney transplant patients, with careful consideration of immunosuppressive status and infection risk. Current evidence supports the relative safety of anti-TNF agents, anti-integrins, and ustekinumab in this setting, although long-term studies are needed to better define their impact on graft function and patient outcomes. Multidisciplinary collaboration among transplant specialists, dermatologists, and rheumatologists is essential to optimize treatment strategies and ensure renal safety in this vulnerable population.

### 5.4 Infection risk and its reciprocal impact on renal function

Biologic agents used in psoriasis and other autoimmune diseases have revolutionized treatment but carry inherent infection risks that may adversely affect renal function. The immunomodulatory effects of biologics, particularly those targeting TNF-α, IL-17, and IL-23 pathways, can predispose patients to infections, which in turn may exacerbate or precipitate kidney injury. In older adults with psoriasis, infections are the most frequently reported adverse events during biologic therapy, although no direct significant association with age was found (85). Nonetheless, older age itself is linked to renal function deterioration in patients using certain systemic agents like cyclosporin, underscoring the complex interplay between infection susceptibility, immunosuppression, and renal outcomes.

In kidney transplant recipients, who often receive biologic or immunosuppressive therapies, infections such as cytomegalovirus, BK virus, and bacterial urinary tract infections (UTIs) are common and significantly impact graft function. Delayed graft function (DGF) is associated with increased risks of BK viremia and UTIs, which can further compromise renal allograft survival (86). Moreover, opportunistic infections remain a major concern in transplant recipients with immunosuppression, as demonstrated by increased incidences of pulmonary aspergillosis, pneumocystosis, and CMV colitis, which correlate with baseline renal function and inflammatory markers (87). The expression of viral cytokine homologs, such as human cytomegalovirus IL-10 transcripts, may also precede viral DNA replication, suggesting that early viral reactivation could be detected and managed to prevent renal damage (88).

The risk of infection and its renal consequences necessitate vigilant monitoring and multidisciplinary collaboration. In spinal cord injury patients, adherence to neuro-urological monitoring guidelines significantly reduces infection risk and preserves renal function, illustrating the importance of systematic follow-up (89). Similarly, in pediatric kidney transplant recipients, viral infections substantially affect graft function and patient outcomes, emphasizing the need for early diagnosis and tailored management (90). The use of induction agents such as thymoglobulin (anti-thymocyte globulin) in kidney transplantation, while effective, carries infection risks that must be balanced against rejection prevention, particularly in low immunological risk patients (91, 92).

In patients with lupus nephritis, infections are independent risk factors for poor renal prognosis, with peripheral blood neutrophil-lymphocyte and lymphocyte-monocyte ratios serving as biomarkers correlating with renal function and infection status (93). The immunosuppressive regimens combining biologics and conventional agents require careful consideration to minimize infection-related renal impairment (94).

The COVID-19 pandemic has further highlighted the bidirectional relationship between infections and renal function. COVID-19 can directly cause AKI and exacerbate CKD, while patients with pre-existing renal impairment have worse COVID-19 outcomes (95, 96). Kidney transplant recipients infected with SARS-CoV-2 exhibit increased risks of AKI and graft function decline, necessitating close surveillance (97). Additionally, COVID-19 infection may induce rare but severe complications such as intrarenal arterial thrombosis in transplant recipients, further jeopardizing graft survival (98).

Given these complexities, prudent monitoring of infection markers, renal function, and immunosuppressive drug levels is essential. Multidisciplinary collaboration among dermatologists, nephrologists, infectious disease specialists, and transplant teams is critical to optimize patient outcomes. Early detection of infections, individualized immunosuppressive regimens, and patient education on infection prevention are vital components of comprehensive care. This approach ensures that the benefits of biologic therapies in managing psoriasis and other autoimmune diseases are not undermined by infection-related renal complications.

## 6 Safety monitoring and risk management of biologic agents

The safety monitoring and risk management of biologic agents, particularly in the context of their renal effects, require a comprehensive and dynamic approach to ensure patient safety and optimize therapeutic outcomes. Establishing a dynamic renal function monitoring system during biologic therapy is fundamental. This system should incorporate regular and systematic assessment of renal parameters such as serum creatinine, eGFR, urinalysis for proteinuria or hematuria, and other biomarkers indicative of renal injury or dysfunction. Given that biologics can modulate immune responses and potentially induce adverse effects including nephrotoxicity or immune complex-mediated glomerulonephritis, continuous monitoring allows for early detection of renal impairment, facilitating timely intervention and adjustment of therapy. The integration of patient-reported outcome measures (PROMs) into monitoring frameworks has been advocated to enhance the detection of ADRs related to biologics, enabling clinicians to capture patient perspectives and subtle changes that might precede clinical manifestations (99). Furthermore, the use of telemedicine and remote biological signal monitoring systems has demonstrated feasibility and safety in other chronic disease contexts, such as cardiac rehabilitation, suggesting potential applicability to biologic therapy monitoring to improve accessibility and adherence to monitoring protocols (100).

Risk assessment models and early warning systems represent another crucial component of safety management. These models should integrate clinical, laboratory, and demographic data to stratify patients according to their risk of developing renal adverse events during biologic therapy. Machine learning and advanced data analytics have been increasingly applied in biosensor technologies and risk assessment frameworks, enhancing the sensitivity and specificity of monitoring systems for biological risks (101). For biologics, incorporating molecular characteristics, pharmacodynamics, and patient-specific factors into risk assessment algorithms can improve predictive accuracy. For instance, decision trees that incorporate molecular weight and bioavailability parameters have been developed to standardize occupational exposure risk assessments for biologics, which could be adapted to clinical safety monitoring (102). Early warning systems based on real-time data capture and analysis can alert clinicians to deviations in renal function or emerging adverse events, promoting proactive management.

Pharmacovigilance programs play a pivotal role in post-marketing safety surveillance of biologics. Real-world data from pharmacovigilance studies have provided insights into the incidence and nature of adverse events, including renal complications, associated with biologic therapies in various diseases (103). These programs support the identification of rare or unexpected adverse events not captured in clinical trials and inform risk minimization strategies. Moreover, the integration of biological monitoring with environmental and occupational safety assessments underscores the importance of a multifaceted approach to managing biological risks, emphasizing the need for rigorous aseptic techniques and environmental controls during biologic preparation and administration to prevent contamination and infection (104).

Therefore, the safety monitoring and risk management of biologic agents with respect to renal function necessitate a dynamic, multifactorial system that combines regular clinical and laboratory assessments, patient-reported outcomes, advanced risk stratification models, and early warning mechanisms. Leveraging technological advances such as machine learning and remote monitoring can enhance the sensitivity and efficiency of these systems. Continuous pharmacovigilance and adherence to best practices in biologic handling further contribute to minimizing risks and ensuring patient safety during biologic therapy.

## 7 Clinical application prospects

### 7.1 Exploration of novel biologics and combination therapeutic strategies

Novel biologics and combination strategies represent promising avenues for managing psoriasis patients with renal involvement. Emerging agents selectively targeting inflammatory pathways common to both psoriasis and renal pathology, such as IL-17 and IL-23 inhibitors, have shown favorable renal safety profiles (105). Agents like telitacicept, effective in lupus nephritis, a renal complication sharing immunopathogenic features with psoriatic nephropathy, may offer translational potential for psoriatic nephropathy (106).

Combination approaches integrating biologics with conventional therapies have also been explored. Real-world data indicate that combining biologics with methotrexate does not significantly increase adverse events, supporting the safety of such regimens (107). Similarly, adding apremilast to ongoing biologic therapy appears generally safe and may improve disease control without exacerbating renal effects (108, 109). These findings are particularly relevant for patients with renal comorbidities where monotherapy may be insufficient.

Further strategies include dual biologic therapy, such as IL-23 with TNF-α inhibitors, which has shown enhanced efficacy in refractory cases without notable renal toxicity (110). Pediatric populations have also safely utilized combined biologic and conventional therapies for severe disease phenotypes (111, 112). Combinations with phototherapy offer synergistic benefits while maintaining renal safety (113), and in patients with cardiovascular comorbidities, combining biologics with antiplatelet agents may attenuate systemic inflammation and indirectly benefit renal function (114).

Despite these promising developments, the evidence base remains limited, underscoring the need for well-designed clinical trials to establish efficacy and safety in this population. Pharmacogenetic insights may further guide personalized strategies to optimize renal outcomes while minimizing toxicity (115).

### 7.2 Application of multidisciplinary collaboration model in psoriatic kidney management

**Figure 3:**
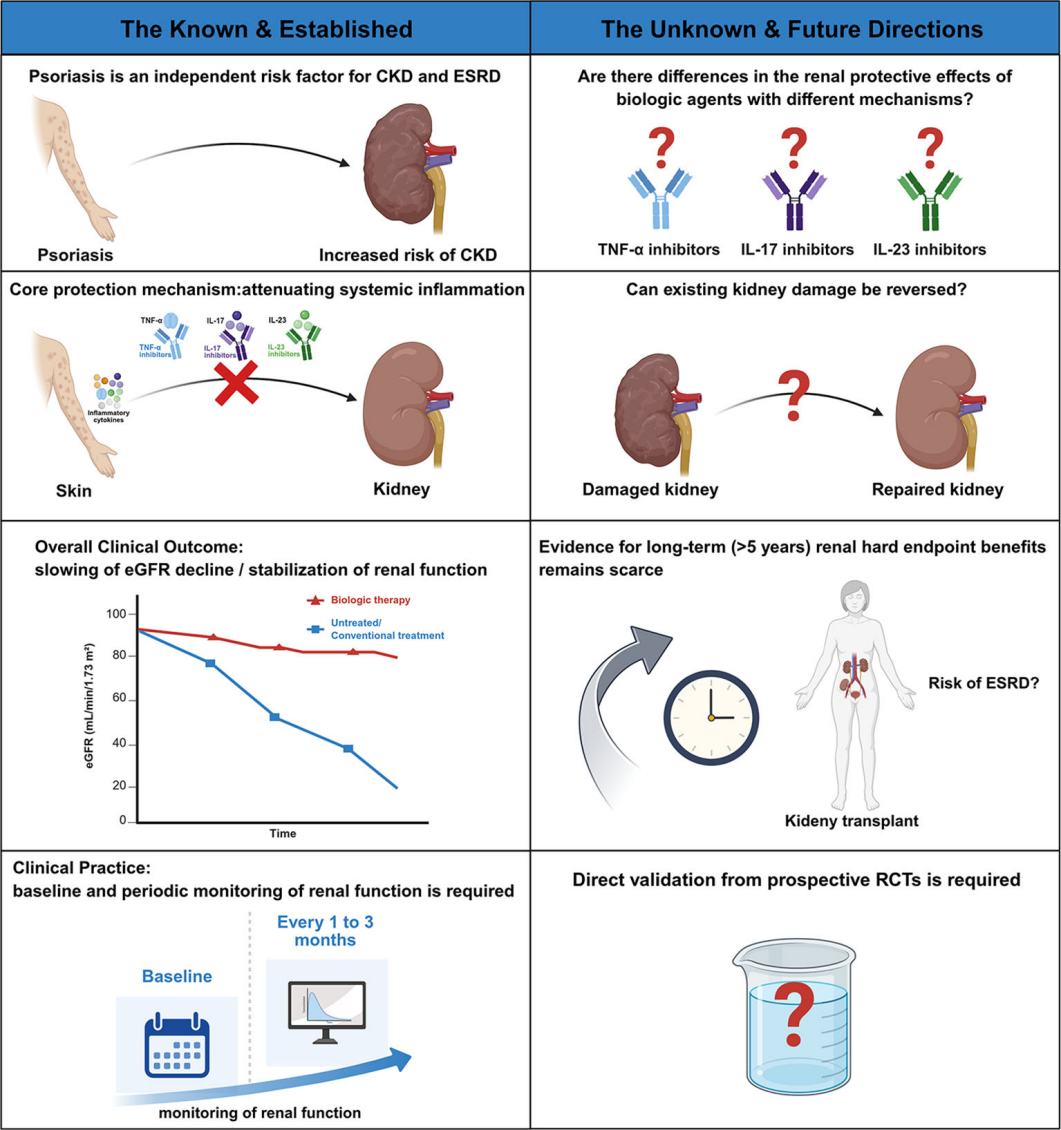
The known and unknown about various biologics on renal function in psoriasis patients. This figure points out the direction for future clinical practice and scientific research work.

Effective management of renal involvement in psoriasis requires a structured multidisciplinary approach bridging dermatology and nephrology. As illustrated in **Figure 3**, this collaboration operates at two levels: implementing established knowledge while jointly exploring unresolved questions.

#### 7.2.1 Translating established knowledge into clinical practice

The left panel of **Figure 3** highlights established concepts that form the foundation of multidisciplinary management. It is now well-established that psoriasis is an independent risk factor for CKD, driven by systemic inflammation. Dermatologists play a central role in recognizing this risk and initiating appropriate referrals. Biologic therapies targeting inflammatory cytokines such as TNF-α, IL-17, and IL-23 have demonstrated efficacy in controlling psoriasis and may influence renal outcomes. However, their use necessitates careful assessment of renal function and potential nephrotoxicity, underscoring the need for close communication between specialties (1, 116). The established goal of therapy is to stabilize eGFR and slow its decline, a target that requires joint monitoring. To achieve this, a practical clinical framework derived from the “known” includes baseline renal assessment and coordinated monitoring.

Furthermore, nephrologists’ input is critical when managing psoriasis patients with advanced CKD or those undergoing dialysis or renal transplantation, as immunosuppressive regimens and biologic agents must be tailored to minimize risks of infection and graft rejection (117, 118). The multidisciplinary approach also extends to addressing systemic inflammation and comorbidities such as metabolic syndrome and cardiovascular disease, which exacerbate renal risk and are prevalent in psoriasis populations (119).

#### 7.2.2 Addressing the unknown through collaborative research

The unresolved questions highlighted in **Figure 3**—differential effects across biologic classes, potential for injury reversal, and long-term renal outcomes—inherently require multidisciplinary investigation. Prospective registries combining dermatologic and nephrologic data can clarify whether specific biologics offer superior renal protection. Long-term follow-up studies are needed to determine if early, sustained control of inflammation translates into reduced ESRD risk. The call for rigorous randomized controlled trials, as emphasized in **Figure 3**, represents a shared priority. Designing such studies requires integrated expertise in trial methodology, psoriasis outcome measures, and renal endpoint adjudication. These collaborative efforts will generate the evidence needed to refine treatment guidelines and optimize outcomes for psoriasis patients with renal involvement.

In summary, a structured multidisciplinary collaboration—grounded in established knowledge and driven by shared questions about the unknown—is essential. By implementing joint monitoring protocols and fostering integrated research, dermatologists and nephrologists can collectively optimize biologic therapy to not only control skin disease but also safeguard long-term kidney health.

## 8 Conclusion

The interplay between psoriasis and renal function impairment presents a complex clinical challenge that demands careful consideration. Psoriasis patients are at a notable risk for kidney dysfunction, a factor that should not be overlooked in comprehensive disease management. The advent of biologic agents has revolutionized psoriasis treatment by targeting specific immune pathways; however, their effects on renal function are multifaceted and warrant nuanced evaluation.

Current clinical evidence largely supports the renal safety of most biologic therapies, with no significant exacerbation of kidney impairment observed in the majority of treated patients. Notably, certain IL-17 inhibitors have demonstrated potential in stabilizing renal function, suggesting a possible nephroprotective role mediated through modulation of immune-inflammatory responses and regulation of cell death pathways. These findings underscore the dualistic nature of biologics: while primarily designed to control cutaneous and systemic inflammation in psoriasis, they may also influence renal pathophysiology, either directly or indirectly.

Nevertheless, the presence of comorbid conditions such as diabetes mellitus and pre-existing kidney disease remains a critical determinant of renal outcomes in this patient population. These risk factors can potentiate renal deterioration irrespective of biologic therapy, highlighting the importance of individualized risk stratification. Balancing the therapeutic benefits of biologics against potential renal risks necessitates vigilant renal function monitoring and a personalized approach to biologic selection. This strategy should be integrated within a multidisciplinary framework involving dermatologists, nephrologists, and other specialists to optimize both psoriasis control and renal preservation.

Despite promising insights, the precise mechanisms by which biologics may confer renal protection remain incompletely elucidated. Further mechanistic studies are essential to unravel the immunological and molecular pathways involved, which could inform the development of targeted interventions to mitigate renal complications. Moreover, there is a compelling need for high-quality prospective clinical trials to validate the safety and efficacy of biologic agents in psoriasis patients with varying degrees of renal impairment. Such evidence will be pivotal in establishing standardized guidelines for the management of this complex comorbidity.

In summary, the evolving landscape of biologic therapy in psoriasis offers a dual opportunity: effective disease control and potential renal protection. Achieving this balance requires a sophisticated understanding of the interplay between systemic inflammation, biologic mechanisms, and renal pathophysiology. Through ongoing research, multidisciplinary collaboration, and personalized clinical strategies, it is feasible to enhance patient outcomes by simultaneously addressing cutaneous disease activity and safeguarding renal function. This integrated approach represents the future direction for optimizing care in psoriasis patients at risk for or experiencing renal impairment.
